# Graphic angle measure as an electrocochleography evaluation parameter

**DOI:** 10.1590/S1808-86942011000200011

**Published:** 2015-10-19

**Authors:** Karen de Carvalho Lopes, Mário Sérgio Lei Munhoz, Marco Aurélio Rocha Santos, Márcio Flávio Dutra Moraes, Adriana Gonzaga Chaves

**Affiliations:** 1 Specialized in Neurotology - Federal University of São Paulo / Paulista School of Medicine, MSc in Sciences - Department of Otolaryngology and Head and Neck Surgery - Federal University of São Paulo / Paulista School of Medicine - ENT physician; 2 Associate Professor of Otorhinolaryngology. Adjunct Professor of Otorhinolaryngology - Department of Otolaryngology and Head and Neck Surgery - Federal University of São Paulo / Paulista School of Medicine; 3 PhD in Sciences - Federal University of São Paulo / Paulista School of Medicine. ENT physician - Medical School - Federal University of Minas Gerais; 4 PhD in Sciences - Medical School of Ribeirão Preto / University of São Paulo. Professor - Department of Physiology and Biophysics - Federal University of Minas Gerais; 5 MSc in Sciences - Department of Otolaryngology and Head and Neck Surgery - Federal University of São Paulo / Paulista School of Medicine - ENT physician. Departamento de Otorrinolaringologia e Cirurgia de Cabeça e Pescoço da Universidade Federal de São Paulo - Escola Paulista de Medicina, São Paulo SP Brasil e Setor de Audiologia do Anexo São Geraldo do Hospital das Clínicas da Universidade Federal de Minas Gerais Belo Horizonte MG Brasil

**Keywords:** endolymphatic hydrops, meniere disease, audiometry, evoked response

## Abstract

To improve electrocochleography's diagnostic sensitivity in Meniére's disease - new assessment methods are being studied.

**Aim:**

To determine whether or not graphic angle measurement is sensitive and specific to Menière's disease laboratorial diagnosis and if there is an increase in the electrocochleography's sensitivity and specificity when graphic angle measurements are associated with Summating Potential-Action Potential ratio (SP/AP ratio).

**Methods:**

Electrocochleography's was used to analyze 71 ears from 55 subjects: 41 patients with clinical diagnosis of Menière's disease (MD group), and 14 healthy individuals as control (Group C). Graphic results were analyzed initially to obtain the SP/AP ratio; afterwards, through another program graphic angle measurements were calculated.

**Results:**

Sensitivity and specificity values of angle measures, SP/AP ratio, and the association between them varied according to the cutoff point, the highest equilibrium between sensitivity and specificity was observed with the values of 166.25 for angle measurement and 27% for SP/AP relation; 62.79% / 60.71% and 74.42% / 67.86%, respectively. The association between measurements showed a sensitivity increase due to the specificity decrease; 88.37% and 50%, respectively.

**Conclusion:**

Angle graphic measurement is not sensitive and specific enough for the laboratorial diagnosis of MD. Angle graphic measurement and SP/AP ratio association proved to be higher in sensitivity, in detriment of exam specificity.

## INTRODUCTION

In 1848, Prosper Ménière was the first to report an association between vertigo and labyrinthine disease. In 1871, Knapp suggested the idea of increased intracochlear pressure. It was only in 1938, that Hallpike and Cairns described, based on a histopathology slide of the temporal bone, the change which is broadly known today: the dilatation of the endolymphatic system, and used the term Endolymphatic Hydrops (EH) to characterize such finding^1^, ^2^.

When the cause of EH is not found, it is then called Ménière's Disease (MD). Some conditions such as: infections, trauma, otosclerosis, syphilis, genetic causes, allergies, electrolytic and metabolic disorders, and autoimmune diseases are associated with the development of EH^3^, ^4^, ^5^, ^6^, ^7^.

The Balance and Hearing Committee of the American Academy of Otorhinolaryngology and Head and Neck Surgery (AAO-HNS) published the criteria they use to clinically diagnose Ménière's Disease (MD), in which the patients are classified as: defined MD, probable MD or possible MD. MD is considered defined in the presence of two or more spontaneous vertigo spells lasting 20 minutes or more, associated with documented sensorineural hearing loss in at least one occasion, ear fullness and tinnitus. It is called probable when there is one defined vertigo spell in the presence of documented sensorineural hearing loss in at least one occasion, ear fullness or tinnitus. And it is classified as possible when there is episodic Ménière's type of vertigo without documented hearing loss or when there is fixed or floating sensorineural hearing loss associated with unbalance, without a defined episode of vertigo. Certainty in the diagnosis of endolymphatic hydrops is only possible by means of a *post mortem* histopathology study of the temporal bone.^8^

Despite AAO-HNS recommendations, there has been a tendency towards ordering objective diagnostic tests in order to corroborate the diagnosis of this disorder, including electrocochleography (EcochG) and electronystagmography^9^.

The electrical signs recorded from EcochG are the reflex of numerous ionic currents associated with the process of transduction in the cochlear hair cells and with the action potential generation on the cochlear nerve fibers: cochlear feedback (MC), summation potential (SP) and the action potential (AP)^10^, ^11^, ^12^, ^13^. EcochG has become one of the first and few electrophysiological measures of MD.

In order to mitigate the large interindividual variability concerning the amplitude of responses, a ratio between the SP and the AP was introduced, known as the SP/AP ratio, making the SP assessment safer, which is important for the diagnosis of EH^14^, ^15^.

In a recent publication^16^, the authors described the Graphic Angular Measure (GAM), a technique which expresses the variability in amplitude, latency and wave skew, by measuring one angle. The individuals examined were submitted to audiometric evaluation and to extratympanic electrocochleography. The author of this paper did not characterize the type of MD affecting the group studied; he only describes the technique and shows normative values.

The lack of studies with this technique, the lack of information with the use of the transtympanic electrode, its simplicity and the possibility of association with other interpretation parameters motivated us to carry out the present study.

The goal of this paper is to study whether the graph angular measure is sensitive and specific for the electrophysiological diagnosis of MD, and to assess whether or not there is an increase in sensitivity and specificity of electrocochleography for the objective diagnosis of MD when the graphic angular measure is associated with the value of the SP/AP ratio.

## METHODS

This is a multicentric cross-sectional cohort study which was approved by the Ethics Committees of the Institutions where the study was carried out, according to the following protocols.

71 ears from 55 individuals from both genders, with ages varying between 18 and 75 years of age were included in the study. As far as the age of the sample is concerned, between the two research centers, we setup only minimum and maximum values for age, and we included only those patients who matched the inclusion criteria and were within this age range. The same thing was done for control individuals.

All patients and controls were submitted to otorhinolaryngological exam, tonal and vocal audiometry, immittance measurements and bilateral transtympanic electrocochleography (EcochG TT). We took off those patients with neurological disorders, neoplasia, otitis, tympanic membrane perforation, a past of head injury or ear surgery, and those who did not sign the free and informed consent form.

The individuals were distributed in 2 study groups:

MD GROUP (GDM): 43 ears from 41 patients, 7 men and 34 women, with a clinical defined diagnosis of MD, according to the 1995 AAO-HNS criteria, with normal otoscopy and bilateral type A tympanometric curves.
CONTROL GROUP (GC): 28 ears from 14 individuals, 6 men and 8 women, all volunteers without neurotological or neurological symptoms, normal otoscopy exam, audiometric tonal thresholds below 25 dB hearing level (dBHL) in all frequencies studied, type A bilateral tympanometric curves.

All EcochG TT were carried out using the Navigator SE® device from *Bio-logical Systems Corp.* The tympanic membrane was seen by means of a surgical otoscope and done under topical anesthesia with 10% xylocaine spray. We used chlorinated silver surface leads, placed on the posterior surface of the ear lobes. One periauricular support was placed over the ear to be examined and it was fixed by means of a head band, with the aim of keeping the transtympanic electrode in place. We then positioned the transtympanic electrode on the postero-inferior quadrant of the tympanic membrane, near the cochlear window niche. We used a Teflon-coated needle-shaped monopolar electrode, 4 centimeters long and one and a half millimeter thick.

The electrocochleography exams were carried out by one examiner from each participating center, using the same type and brand of device, with the same specifications and following the same technical parameters. The examiners knew who the patients were and who the controls were, since the latter were healthy volunteers and sometimes, people they knew.

The recordings followed the setup: transtympanic electrode = not inverted (active), ipsilateral lobe = inverted (reference), contralateral lobe = ground; according with the protocol:

**Table:** 

Stimulus	click
Stimulus duration	100 *μ*s
Polarity	Alternate
Presentation pace	7.1 stimuli per second
Intensity	90 dB hearing level
Masking	off
Quantity	300 stimuli per recording
Recording repetitions	two
High pass filter	1.500 Hz
Low pass filter	3 Hz
Notch filter	off
Gain	50.000
Analysis window	10,24 ms
Transducer	TDH39

### Graph angular measure calculation

After identifying the BSL, the SP and the AP, the traces were initially analyzed by the equipment software in order to obtain the SP/AP ratio. Later on, the SP and AP latency and amplitude data, in relation to the baseline, were analyzed by another software in order to obtain the necessary lines to be used to calculate the angle, which was done by means of the X and Y coordinated (Excel® spreadsheet, created by one of the authors of the present paper, who is an electronic engineer). The angle was formed by the intersection of two lines, one drawn perpendicular to the SP and the other traced in such a way as to connect the two peaks: SP and AP.

The mathematical formula used to do the angle calculation was inserted in an Excel® spreadsheet, where the latency and amplitude (v) values were calculated (ms) for SP and AP, where: Delta V = PA(v) - PS(v) and Delta T = PA(ms) - PS(ms).

The formula was given by: ANGLE= ARCTAN (Delta V / Delta T) x 180 / ϖ + 90.

**Figure 1:**
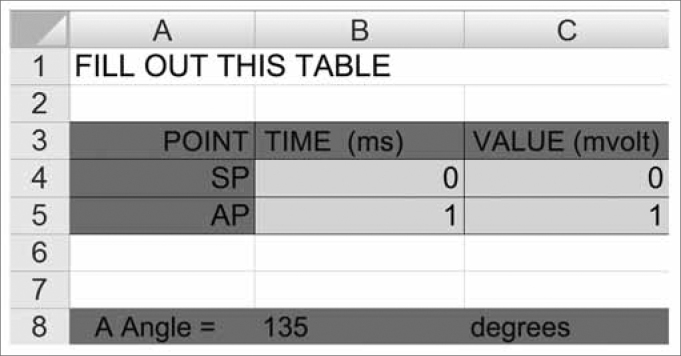
Excel® spreadsheet used to calculate the angular measure. After filling out the green fields with the latency and amplitude values we obtain the angle value.

**Figure 2:**
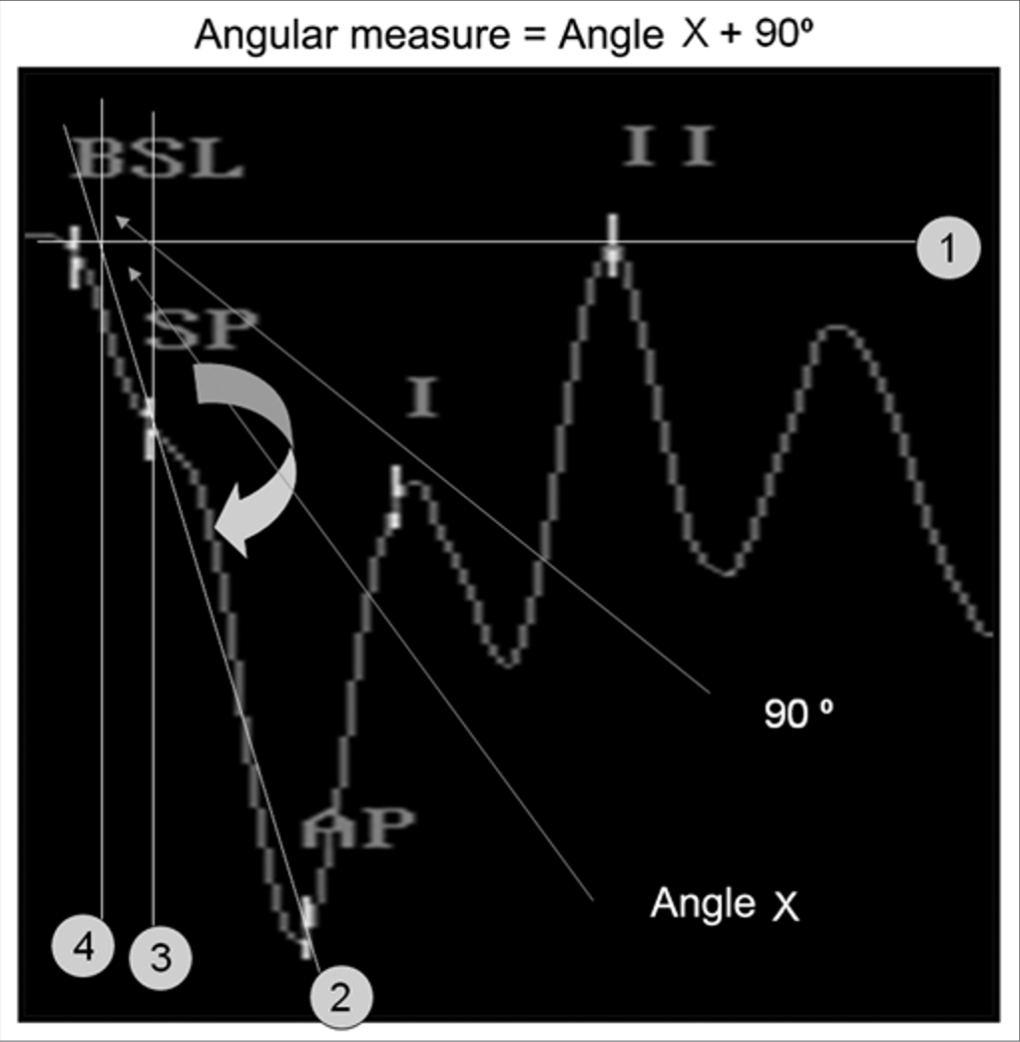
Steps to obtain the graphic method in order to measure the angle:
-Place line 1 on the baseline,-Place line 2 between SP and AP and extend it all the way to the baseline,-Place line 3, which passes through the SP and is perpendicular to the baseline,-We then obtain the Angle which will be measured.-Place line 4 parallel to line 3 perpendicular to the baseline and on the point where line 2 meets the baseline,-Here we have the X angle-The measure corresponds to the X angle plus 90°-The X angle is calculated in the formula with the x and y coordinate points in the plane, latency and amplitude in reference of the baseline.

Where ARCTAN is the arch which tangent is equal to (DeltaV / DeltaT). Since this arch was expressed in radians, in order to convert it into degrees it was necessary to use the conversion constant: 180/ϖ, where ϖ= 3.1415.... because our object of interest was the measurement in relation to the vertical axis, it was necessary to add 90^0^ to the obtained value (Figures 1 and 2).

The SP marking standardization was the point of the highest amplitude, or, should it be absent, on the first trace deflection, after the baseline. The SP and AP marks in the trace were made only by the examiner (one in each center) who had carried out the exam. The angular measure calculation, using the spreadsheet, was carried out only by the main researcher.

### Statistical analysis

The data analysis was carried out with the SPSS statistical package (*Statistical Package for Social Sciences*) *for Windows*, version 14.0. All the tests were carried out considering bilateral hypothesis and assuming a significance level of α=5%.

We initially used descriptive statistics to assess the frequency, mean and standard deviation of the variables of interest. We used the chi-square, t-test and Fisher's Exact test in order to study the variables.

We used the areas under the ROC (*Receiver operating characteristic*) curve in order to obtain accuracy, sensitivity and specificity for the angular measure, the SP/ AP ratio and the two measures together, considering the GC and the GDM. The ROC curve is a graphic line which depicts the likelihood of a truly positive result - or the test sensitivity - *versus* the likelihood of a false positive result for a number of different points in the cutting point. In the graph, the closer the line is to the upper left-hand corner of the graph, the more accurate the test is. Moreover, the point closer to this corner is usually chosen as the cutting point which simultaneously maximizes sensitivity and specificity.

## RESULTS

On Table 1 we notice a mildly higher proportion of women in the GDM (82.9) when compared to the GC (57.1); this difference is not statistically significant (*p*=0.071). The same happens with the “ear side” variable (*p*=0.774). Thus, GC and GDM are homogeneous according to gender and ear side.

**Table 1:** Frequency and percentage distribution of the ears allocated to groups GC and GDM according to gender and the side of the ear involved.

		GC N (%)	GDM N (%)	*p*-value*
Gender+	Male	6 (42,9)	7 (17,1)	0,071
	Female	8 (57,1)	34 (82,9)	
Ear side*	Right	14 (50,0)	20 (46,5)	0,774
	Left	14 (50,0)	23 (53,5)	

* Pearson's chi-square; +Fisher's Exact Test

We noticed on Table 2 that GC has a lower mean age when compared to GDM (*p*<0.001).

**Table 2:** Mean, Standard Deviation and *p*-value for age comparisons in GC and GDM.

	GC Mean (s.d)	GDM Mean (s.d.)	*p*-value*
Age	33,07 (11,13)	49,78 (11,96)	<0,001*

* t-test

We noticed on Table 3 that there is a significant difference between GC and GDM for the SP/AP ratio (*p*<0.001) and angular measure (*p*=0.016).

**Table 3:** Standard Deviation and *p*-value for comparing the means for the SP/AP ratio values and angular measures of GC and GDM.

	GC	GDM	*p*-value
	Mean (s.d)	Mean (s.d.)	
SP/AP	0,25 (0,07)	0,37 (0,12)	<0,001*
Medida Angular	165,30 (9,23)	156,03 (21,72)	0,016*

* t-test

**Figure 3:**
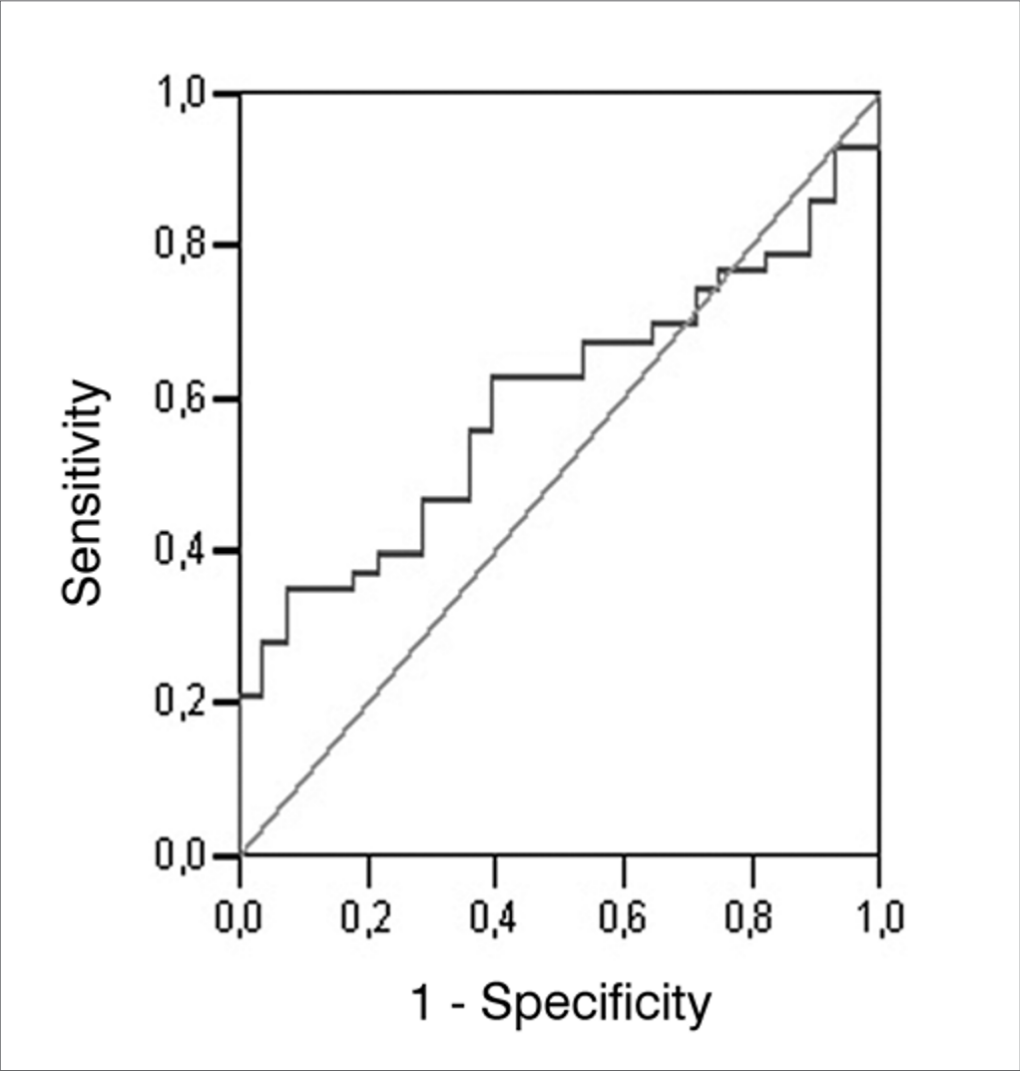
Graphic representation (ROC curve) of the plotting of numerous sensitivity and specificity points of the angular measure considering GC and GDM.

**Figure 4:**
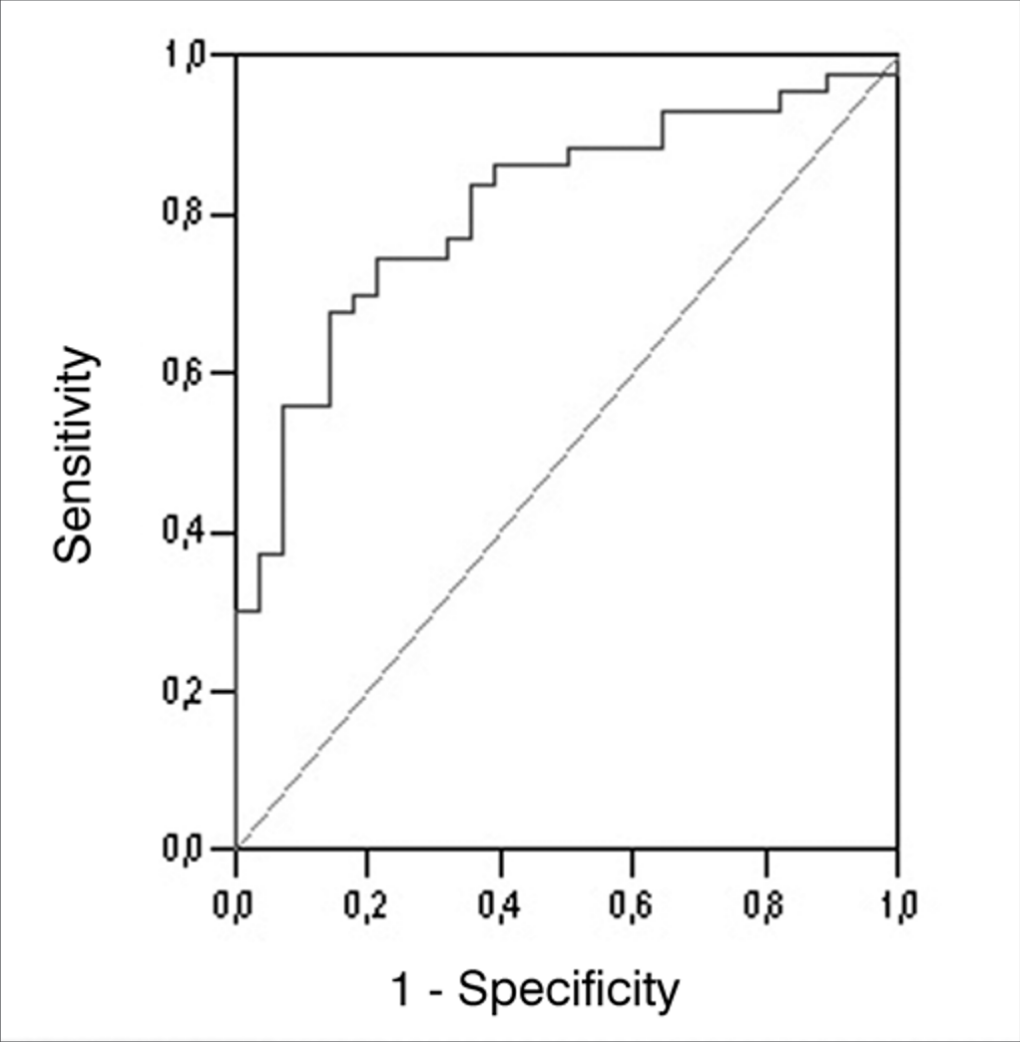
Graphic representation (ROC curve) of the plotting of numerous sensitivity and specificity points of the SP/AP ratio considering GC and GDM.

**Figure 5:**
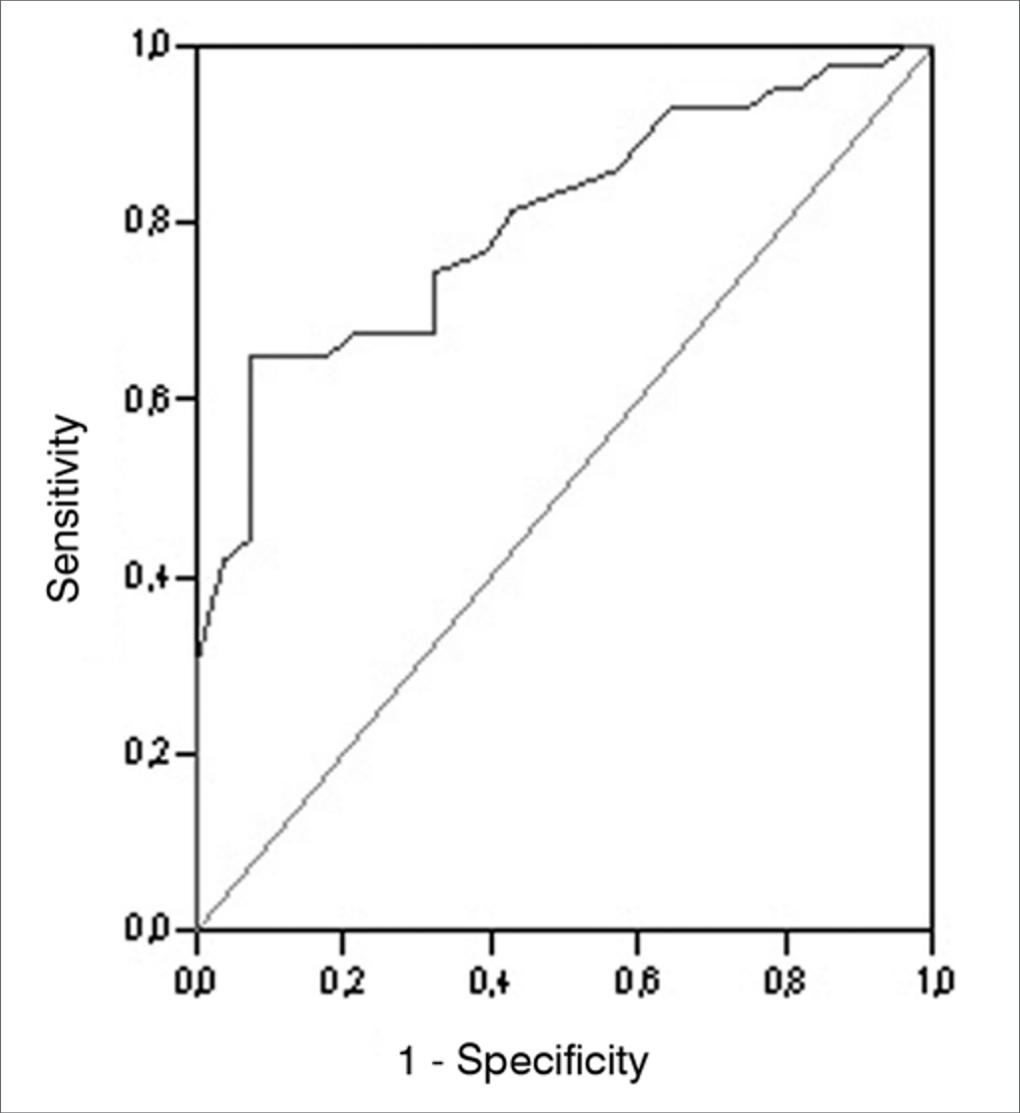
Graphic representation (ROC curve) of the plotting of numerous sensitivity and specificity points of the angular measure and SP/ AP ratio combination considering GC and GDM.

The values corresponding to the areas below the ROC curve, the cutting points, the sensitivity and specificity values are depicted on Table 4; the angular measure variable on Table 5, considering the SP/AP ratio; and on Table 6, the two variables together. The ROC curves are presented on Figs. 3, 4 and 5.

**Table 4:** Área sob a curva ROC, valores de corte e respectivos valores de sensibilidade e especificidade da medida angular para os GC e GDM.

Variable	Area (CI 95%)	*p*-value	Cutting Score	S (%)	E (%)
			166.15	60.47	60.71
			166.25	62.79	60.71
			166.86	62.79	57.14
Angular Measure	0.596 (0.466 - 0.727)	0,172	167.86	62.79	53.57
			168.33	62.79	50.00
			168.52	62.79	46.43
			168.71	65.12	46.43

S: sensitivity, E: specificity

**Table 5:** Area under the ROC curve, cutting scores and respective sensitivity and specificity values for the SP/AP ratio for GC and GDM.

Variable	Area (CI 95%)	*p*-value	Cutting Score	S (%)	E (%)
			0.23	93.02	35.71
			0.24	86.05	42.86
			0.25	81.40	57.14
SP/AP	0.802 (0.702 - 0.903)	<0.001	0.26	76.74	60.71
			0.27	74.42	67.86
			0.28	72.09	67.86
			0.29	67.44	67.86

S: sensitivity, E: specificity

**Table 6:** Area under the ROC curve, cutting scores and respective values for sensitivity and specificity concerning the combination of the angular measure and the SP/AP ratio for GC and GDM.

Variable	Área (IC 95%)	*p*-value	Cutting score	S (%)	E (%)
			-0.56	93.02	35.71
			-0.55	90.70	35.71
			-0.50	88.37	35.71
Angular Measure and SP/AP	0,807 (0,706 - 0,909)	<0,001	-0.44	88.37	39.29
			-0.39	88.37	42.86
			-0.37	88.37	46.43
			-0.36	88.37	50.00

S: sensitivity, E: specificity

We chose the cutting scores which presented the highest balance between sensitivity and specificity: 166.25 for the angular measure, 0.27 for the SP/AP ratio and -0.36 for the association of both measures. We observed on Table 7 that there was an increase in the number of properly classified sick patients when we use the measures together (88.4%); the SP/AP ratio alone is better than the angular measure to diagnose patients with MD.

**Table 7:** Frequency and percentage distribution of patients properly classified according with the cutting values for each measure, chosen from the ROC curve.

Test	Number of properly classified sick patients	Percentage of properly classified sick patients
Angular measure	27	62.8
SP/AP	31	72.1
Angular measure and SP/AP	38	88.4

## DISCUSSION

The SP/AP ratio, although broadly spread, has a low sensitivity between spells, which is especially problematic for patients with uncertain clinical profiles. It is important, therefore, to have studies with would target to increase EcochG sensitivity for the laboratorial diagnosis of MD in patients out of the active stage of the disease. One problem with the development of objective tests for the diagnosis of MD is the lack of a gold standard^17^.

Concerning demographic characteristics, in our study we observed a higher prevalence of females in the group of patients with MD (82.9%). This finding was similar to those reported by Chung et al.^18^ and Chaves et al.^19^, with prevalences of 61.4% and 79.5%, respectively, and also corroborated by studies by Levine et al.^11^ and Pappas et al.^20^.

The mean age found in the group of patients was 49.78, varying between 19 and 73 years, similarly to what was observed in other studies^18^, ^19^, ^20^, ^21^, ^22^.

The mean value of the SP/AP ratio proved to be statistically different between both groups, and there was a correlation between the two values, with a trend for both groups to present, for values lower than the SP/AP ratio, higher values of the angular measure and vice-versa.

Sensitivity was considered as the proportion of truly positive values in patients with MD and specificity as the proportion of truly negative responses in patients with normal ears.

In the pertaining literature, the sensitivity values for ECochG, when interpreted only by the SP/AP ratio value, vary substantially, 28%^11^, 40%^23^, 52.4%^24^, 70%^25^, 76.1%^26^, 80%^27^. A mean value of 60% is accepted during intervals without MD spells. Some explanations for this variability include patient's hearing level, stimulation used, cutting score for a positive exam, measure characteristics and lead location^28^. In our study, we analyzed groups C and MD, the value of the SP/AP ratio was 0.27, and it was the one which showed the highest balance between sensitivity and specificity (sensitivity of 74.42% and specificity of 67.86). The choice of the SP/AP ratio value of 0.27 was based on the highest balance point between the cutting scores plotted on the ROC curve - one attempt to simultaneously maximize sensitivity and specificity. The sensitivity and specificity values may be higher or lower, depending on the test's purpose. In theory, the best thing is that the test be highly sensitive and specific. Nonetheless, this is usually not possible. Many of these tests are really based on a clinical measure which may take on a number of values, should that be the case, there is an inherent component between sensitivity and specificity. Similarly to our study, Kim et al.^29^ analyzed the sensitivity and specificity in relation to the SP/AP ratio cutting value and noticed that when the value of this fraction is high, the ECochG's specificity in detecting HE is increased in detriment of sensitivity.

Analyzing the C and MD groups, the 166.25 angle value was the one which showed the highest balance between sensitivity and specificity (62.79% sensitivity and 60.71% specificity), both values were below those of the SP/PA ratio. We noticed that for higher values there is an increase in the sensitivity, in detriment of specificity; this happens because of the low test accuracy, making it bad to differentiate normal individuals for diseased ones.

In this study, as we analyzed the sensitivity and specificity of the combination between the SP/AP ratio and the angular measure, we noticed an increase in sensitivity (88.37%), in detriment of specificity (50%). This could cause a reduction in the number of false negative tests; however, an increase in the number of false positives. Even then, this association was the one which had the highest percentage of properly classified sick patients (88.4%), when compared to the SP/AP ratio (72.1%) and angular measures (62.8%) alone. Similarly, Ikino & Almeida^24^ studied that AP latency difference and amplitude x width index and concluded that, despite being significantly higher in the MD group, these parameters did not increase the EcochG's sensitivity in the HE diagnosis.

We stress that a high sensitivity indicates that the test can be used to rule out a disease when it is negative, and a high specificity is useful to confirm the disease when the test result is positive.

## CONCLUSION

The graph angular measure is not sensitive, nor specific enough for the laboratorial diagnosis of MD.

The association of the graphic angular measure and the SP/AP ratio has the better sensitivity, despite the specificity of the diagnostic test, when compared to the measures alone.
